# PhyME: A probabilistic algorithm for finding motifs in sets of orthologous sequences

**DOI:** 10.1186/1471-2105-5-170

**Published:** 2004-10-28

**Authors:** Saurabh Sinha, Mathieu Blanchette, Martin Tompa

**Affiliations:** 1 Center for Studies in Physics and Biology, The Rockefeller University, New York, NY 10021, USA; 2 School of Computer Science, McGill University, 3480 University Street, Montreal, QC, H3A 2A7, CANADA; 3 Department of Computer Science and Engineering, University of Washington, Seattle, WA 98195-2350, USA

## Abstract

**Background:**

This paper addresses the problem of discovering transcription factor binding sites in *heterogeneous *sequence data, which includes regulatory sequences of one or more genes, as well as their orthologs in other species.

**Results:**

We propose an algorithm that integrates two important aspects of a motif's significance – *overrepresentation *and *cross-species conservation *– into one probabilistic score. The algorithm allows the input orthologous sequences to be related by any user-specified phylogenetic tree. It is based on the Expectation-Maximization technique, and scales well with the number of species and the length of input sequences. We evaluate the algorithm on synthetic data, and also present results for data sets from yeast, fly, and human.

**Conclusions:**

The results demonstrate that the new approach improves motif discovery by exploiting multiple species information.

## Background

The discovery of novel transcription factor binding sites in regulatory sequences of genes has been an important scientific challenge for some years now. Computational approaches to this problem have come in two flavors. One class of methods looks for overrepresented motifs in sequences that are believed to contain several binding sites for the same factor (such as promoters of co-regulated genes) [1-6]. The second class of methods identifies motifs that are significantly conserved in orthologous sequences, e.g., promoters of the same gene in different species [7,8]. These two approaches have been applied to their respective kinds of data sets, with moderate success. However, with new genomes being sequenced regularly, motif-finding applications today often present *heterogeneous *sequence data that includes promoters/enhancers of multiple co-regulated genes in one species, as well as their orthologs in other species. This paper presents a probabilistic algorithm, called "PhyME" (**Phy**logenetic **M**otif **E**licitation), for *ab-initio *detection of binding site motifs in such heterogeneous sequences.

PhyME integrates two different axes of information in evaluating a candidate motif's significance. One axis is that of *overrepresentation*, which depends on the number of occurrences of the motif in each species. The other axis is the level of *conservation *of each motif instance across the species. A real motif that is not sufficiently significant along any one axis may turn out to be significant when both axes are considered simultaneously, leading to increased sensitivity and specificity of the integrated approach. Given the regulatory regions of potentially co-regulated genes along with their orthologs from other species, PhyME uses an Expectation-Maximization (E-M) algorithm to search for the motif that best explains the data. When evaluating a motif, its orthologous occurrences are assumed related to each other by a probabilistic model of evolution that takes into account the varying phylogenetic distances among the species. (The species may be related by any user-specified phylogenetic tree.) Each E-M iteration scales linearly with the total length of the input sequences and also with the number of species. The algorithm can also handle cases where the heterogeneous data is *incomplete*, i.e., where the orthologous regulatory regions are missing from some species. This capability makes it particularly suitable for applications that include data from incomplete genomes, or where orthology information is incomplete.

An important feature of PhyME is that it allows motifs to occur in (evolutionarily) conserved as well as unconserved regions in orthologous promoters, treating the two kinds of occurrences differently when scoring a motif. It does not require each binding site occurrence in one promoter to have an orthologous occurrence in any or all other species. As a result, PhyME affords some flexibility in terms of the evolutionary distances spanned by the input sequences. For instance, using a distantly related ortholog will help pinpoint motifs located in conserved regions but will not hamper the discovery of motifs absent from that ortholog.

### Comparison with previous work

Traditionally, motif finding algorithms have treated input sequences as being independently generated, and searched for statistically overrepresented motifs in them. These algorithms [1-6] do not have the notion of sequence orthology built into them, and are therefore typically run on sequences from the same species. PhyME has an obvious advantage over them, since it takes motif conservation into account. (Henceforth, *conservation *of motifs will be assumed to mean conservation across species.)

Another class of motif-finding methods take as input sets of orthologous sequences, either aligned [8] or unaligned [7] and search for well-conserved motifs. These methods however, unlike PhyME, do not exploit the other important aspect of a motif's significance – that of overrepresentation.

Some algorithms [9,10] take as input a heterogeneous pool of co-regulated and orthologous promoters, and find overrepresented motifs after treating all sequences (including orthologous ones) as independent. However, this "homogenizing" strategy has its disadvantage, since it treats orthologous (and hence, directly related) motif occurrences as statistically independent observations. PhyME, on the other hand, respects the distinction between orthologous and co-regulated motif occurrences.

There are algorithms that attempt to handle the two axes of information by a two-step approach. For instance, Cliften *et al*. [11] and Kellis *et al*. [12] find a set of highly conserved motifs (in yeast promoters) in the first step, and then extract overrepresented ones from this set, in a second step. The algorithm CompareProspector [13] takes a Gibbs-sampling approach to find overrepresented motifs but biases the search in regions conserved across species. Conversely, one may identify overrepresented motifs in the first step, and then isolate evolutionarily conserved ones among these [14]. In either case, a motif that is relatively weak by either criterion alone, but strong when considering both, may be missed out. PhyME's integrated approach to the heterogeneous data problem addresses this issue. Admittedly, the methods of Cliften *et al*. and Kellis *et al*. have a broader range of applications, since these are genome-wide searches for motifs.

A recent algorithm called orthoMEME (Prakash *et al*. [15]) tackles the heterogeneous data problem by using Expectation-Maximization to search the space of motifs and the space of motif alignments (orthology) simultaneously. Each motif occurrence is assumed to have an orthologous copy in the other species, that could be located anywhere in the corresponding promoter. This is in contrast to PhyME's approach, where orthologous motif occurrences are restricted to pre-aligned regions of the promoters. This restriction comes with the advantage that PhyME scales better with the number of species than does orthoMEME. This is a significant advantage in practice, since the orthoMEME implementation is able to handle only two species data, whereas we have experimented with PhyME on orthologs from up to six species. Moreover, PhyME also allows non-conserved occurrences (those residing outside aligned regions), or occurrences that are conserved in some species and missing in others. Requiring that all motif occurrences come in orthologous sets may be justified for very closely related species, but for more diverged pairs of species (e.g., *D. melanogaster *and *D. pseudoobscura*) the promoters are known to have a mix of conserved and unconserved binding sites [16]. PhyME therefore gains an advantage by looking at both kinds of occurrences. However, orthoMEME's phylogenetic model is more powerful than that of PhyME and can handle a greater range of motif variation than PhyME can.

Our approach is most similar to the algorithm PhyloGibbs (Siddharthan *et al*. [17]), the main differences being that PhyloGibbs (i) uses a Gibbs-sampling approach and (ii) assumes a star topology for the phylogeny, whereas PhyME uses an E-M approach and can handle arbitrary tree topologies. Thus PhyME has a broader domain of applicability in terms of the phylogenetic relationships among input sequences. It may therefore be preferable over PhyloGibbs when the phylogeny is far removed from a star, e.g., in a scenario where a pair of close species is included along with another pair of closely-related species, but the two pairs are greatly diverged from each other. On the other hand, an advantage of using PhyloGibbs is that multiple motifs (for different transcription factors) may be searched in parallel.

The algorithm EMnEM (Moses *et al*. [18]) uses E-M and a phylogenetic model to find motifs, much like PhyME does, except that the former assumes that the input sequences are completely aligned. This assumption may be unsuitable for species at relatively large evolutionary distances, e.g., human and mouse, or *D. melanogaster *and *D. pseudoobscura*. Therefore, PhyME can handle a broader range of species divergence in its input. Another important difference between EMnEM and PhyME is the probabilistic model that each uses to model evolution. While EMnEM is implemented to use the Jukes-Cantor model [19], PhyME uses a more realistic model that incorporates binding site specificities. Thus, in calculating the joint likelihood of aligned motif occurrences, the EMnEM implementation does not use the fact that the effective mutation probability of an ancestral base to some base *β *depends on the fitness of a binding site with *β *at that position. The evolutionary model used in PhyME reflects this dependence, and the incorporation of the model into an Expectation-Maximization framework is one of the main technical contributions of our work. The Results section includes a preliminary comparison of PhyME's performance with that of EMnEM, orthoMEME and PhyloGibbs, on real data.

The algorithm PhyloCon (Wang and Stormo [20]) extends the greedy algorithm of CONSENSUS (Hertz *et al*. [2]) to incorporate multiple species data. However, it treats all orthologous sequences uniformly, ignoring the fact that different species may be at different relative distances from each other. As such, it may be more suitable to use PhyME in cases where the phylogeny is far removed from a star topology of uniform branch length. Also, the PhyloCon algorithm proceeds by first identifying several local multiple alignments in orthologous sequences and then searching for common patterns (motifs) among these multiple alignments. As a result, it may miss motif occurrences that are not well-conserved (or are completely missing) in orthologous sequences. An advantage of PhyloCon is that it does not require the motif length to be input, and instead reports motifs of varying lengths.

## Results

In this section, we first present the new algorithm, and then describe its evaluation on synthetic data, as well as biological data sets from various organisms.

### Algorithm

Suppose that the input includes *n *different promoters (e.g., from co-regulated genes), and for each promoter there are sequences for *K *species. (*K *may be different for different promoters.) PhyME requires that there be one designated "reference species" *σ*_*r *_in the input, for which there is sequence data corresponding to each of the *n *promoters. We shall describe PhyME's algorithm for the special case *n *= 1, though allowing multiple motif instances in this one sequence. The extension to *n *> 1 is trivial, and omitted here for simplicity. Thus, the input consists of a set of sequences *S *= {*S*_1_, *S*_2 _,..., *S*_*K*_}, where *S*_*i *_is the orthologous sequence from species *i*, and one of the *S*_*i*_'s comes from species *σ*_*r*_. The input also includes the motif length *l*, and the phylogenetic tree Ψ over the *K *species, with neutral point mutation rates (probabilities) along each branch. The output is a position weight matrix (PWM) representing the discovered motif, and its score.

PhyME first partially aligns the input sequences and identifies contiguous regions ("blocks") in each 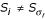 that are highly conserved in 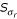. It then inputs all the sequences, along with the locations of the conserved blocks, to the core motif-finding algorithm.

#### Alignment of sequences

**Figure 1:**
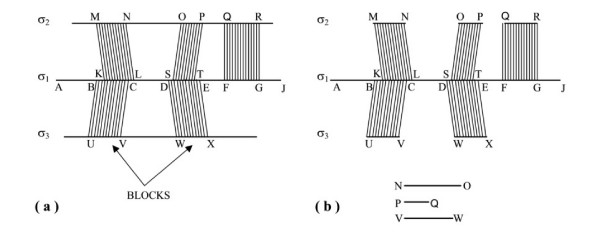
Orthologous promoters and blocks of sequence conservation. Shaded areas represent ungapped aligned blocks. *σ*_1 _is the reference species. (a) Alignment of input sequences and extraction of blocks. (b) Reorganization of input sequences.

In this pre-processing step, PhyME computes the regions of high local similarity between 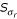 and each of the other *S*_*i*_. The assumption is that such regions are of common evolutionary origin, and any sequence outside them is independently evolved. PhyME runs the LAGAN alignment program of Brudno *et al*. [21] on each sequence pair (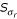, *S*_*i*_|*i *≠ *σ*_*r*_), and extracts all ungapped aligned blocks of a certain minimum size (of the order of the motif's length) and percent identity, to serve as the blocks of common origin. This is illustrated in Figure 1a, which shows orthologous promoters from three species, *σ*_1 _(the reference species), *σ*_2 _and *σ*_3_. An example of a block is region BC in *σ*_1_, aligned with region UV in *σ*_3_. Note how blocks can overlap in the reference species (BC overlaps with KL).

The input is now reorganized into two kinds of sequences:

1. The sequence from the reference species, with aligned blocks of the other species "hanging off" it. (In Figure 1b, this is shown as the sequence AJ, with blocks MN, OP, QR of *σ*_2 _and blocks UV, WX of *σ*_3 _aligned with corresponding blocks in *σ*_1_.) Thus, any position in this sequence either has a single base from the reference species, or has an alignment of bases from multiple species, one of which is the reference species. This entire construct is called the "reference sequence".

2. Any subsequence not from the reference species, and bracketed by blocks on both sides. (e.g., regions NO, PQ, VW in Figure 1b.) The terminal sequences in the non-reference species, which are to the left of the left-most block and to the right of the right-most block, may be optionally included, as per the user's specification.

PhyME fits the parameters of a probabilistic model on the reference and bracketed sequences simultaneously, and the desired motif comes out as a by-product of this training procedure, which is described next.

#### Hidden Markov Model

The probabilistic process that is assumed to generate sequences is described by a very simple *Hidden Markov Model *(HMM). For the moment, let us assume that the sequence *S *being generated is entirely from one species, with no aligned positions. The HMM parameters include a "motif weight matrix" *W*_*m *_of length *l*, and a "background weight matrix" *W*_*b *_of length 1. (The (*k, j*)^*th *^entry of a weight matrix is the probability of emitting the base *j *at position *k *of the sequence being sampled from the matrix.) At each step, the generative process of the HMM chooses either *W*_*m *_or *W*_*b *_according to their *transition probabilities p*_*m *_= *p *and *p*_*b *_= 1 - *p *respectively, where *p *is a model parameter. A sequence is then sampled from the chosen weight matrix, and appended at the end of the sequence *S *created so far. The process then proceeds to the next step. It stops when the length of *S *reaches its known length *L*. The series of motifs chosen in the successive steps of the process is called a "parse" of the sequence. The model parameters *θ*, which include *W*_*m*_, *W*_*b*_, and *p*, associate a well-defined probability *Pr*(*S*, *T*|*θ*) with each parse *T *of the sequence *S*. The probability that *S *was generated by an HMM with parameters *θ *is then given by *Pr*(*S*|*θ*) = Σ_*T*_*Pr*(*S*, *T*|*θ*). Let *Pr*(*S*|*θ*_*b*_) be the probability of generating *S *by using only *W*_*b*_. For a given *θ*, we define


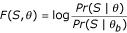


This log-likelihood ratio is the function optimized by PhyME – the parameters *W*_*m *_and *p *are trained so as to maximize *F*(*S*, *θ*). (The background weight matrix *W*_*b *_is not trained during this maximization, rather it is pre-computed from *S*, or optionally from specified background sequence, by measuring nucleotide frequencies.) The value of the objective function for a set of independent sequences is the sum of its values for the sequences taken separately. This additive property allows easy extension of the parameter training procedure to the general case of multiple sequences (*n *> 1). The objective function maximized then is 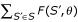, the set *S *now including, for each of the *n *input promoters, the "reference sequence" as well as all "bracketed sequences" (defined earlier) as separate elements.

An important aspect of computing *F*(*S*, *θ*) is the *subsequence probability Pr*(*s*|*W*). This is the probability of generating a subsequence *s *of length *l*, (length of *W*), when sampling from weight matrix *W*; so 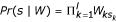, where *s *= *s*_1_*s*_2 _... *s*_*l*_, and *W*_*kj *_is the probability of sampling base *j *at the *k*^*th *^position of *W*. This formula applies when subsequence *s *has a single base at each position. However, we need to adapt this formula to the case where one or more positions in subsequence *s *may be an alignment of orthologous bases from multiple species. In this general case, we can write *s *as *ψ*_1_*ψ*_2 _... *ψ*_*l*_, where each *ψ*_*k *_is either a single base, or an alignment of orthologous bases at a single position of the reference sequence. The subsequence probability *Pr*(*s*|*W*) can then be computed as 
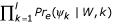
, where *Pr*_*e*_(*ψ*|*W*, *k*) denotes the probability of observing *ψ *at position *k *when sampling from *W*. Let the vector *ψ *= (*s*_1_, *s*_2_, ... *s*_*K*_), where *s*_*σ *_is the nucleotide from species *σ *in the single-base alignment *ψ*. If the *s*_*σ *_were *independent*, we could write 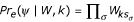. However, the *s*_*σ*_'s occur in an alignment (*ψ*), meaning that this assumption of independence is obviously untenable. Thus we need an expression for *Pr*_*e*_(*ψ*|*W*, *k*) that explicitly takes the phylogenetic relationships among the species (given by the phylogenetic tree Ψ) into account. We present such an expression in the next section, and we shall thereafter return to the topic of maximizing the function *F*(*S*, *θ*).

#### Evolutionary model

This section describes the probabilistic evolutionary model that PhyME uses to incorporate phylogenetic relationships in the computation of the term *Pr*_*e*_(*ψ*|*W*, *k*) mentioned above. It was first proposed in Sinha *et al*. [22] to model binding site evolution, and applied successfully on the two fly genomes. The model makes the crucial assumption that all positions in a binding site evolve independently, at equal rates, and the probability of fixation of a mutation *α *→ *β *at position *k *is proportional to the weight matrix entry of *β *at that position. If we further assume, for simplicity of exposition, that the phylogenetic tree Ψ has a star topology, then the model assumptions give us (from Sinha *et al*. [22]; also see Methods.)


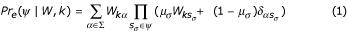


where *s*_*σ *_is the nucleotide from species *σ *in alignment *ψ*, *δ*_*xy *_= 1 if *x *= *y *and 0 otherwise, and *μ*_*σ *_is the neutral mutation probability between the ancestor and the species *σ*. For the position *k*, one "creates" a base *α *in the ancestor with frequency *W*_*kσ*_, and each such base is either passed unchanged to the species *σ *(probability 1 - *μ*_*σ*_) or mutated in species *σ *with probability *μ*_*σ *_and a new base selected with a frequency defined again by *W*.

In the general case, when Ψ does not have a star topology, Formula (1) can be written in a recursive manner. (See Methods.)

#### Expectation Maximization

The function *F*(*S*, *θ*) that is maximized by PhyME measures how much more likely it is that *S *was generated using the motif weight matrix, than without it. Naturally, a PWM that maximizes this score is the motif that best explains the data. PhyME tries to find such a motif by training the parameters (*W*_*m*_, *p*) of the HMM, using the Baum-Welch algorithm [23], which iteratively converges to a locally optimum *θ *using Expectation Maximization (E-M).

Let 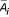, for *i *∈ {*m*, *b*} be the expected number of times the HMM plants motif *W*_*i *_in generating the sequence(s), the expectation being over all parses. Similarly, let 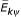 be the expected number of times that the nucleotide alignment *ψ *is sampled at the *k*^*th *^position of the motif *W*_*m*_. 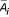 and 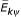 are expected values of *hidden variables *of the HMM. These averages are computed during the "E-step" in each iteration, using dynamic programming (the Forward-Backward algorithm, [23]).

In the "M-step", two kinds of updates are made, using the values of 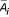, 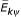 computed in the E-step. The parameter *p *is updated according to 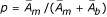. The motif weight matrix *W*_*m *_is updated by solving, for each column *k *of the matrix, the following set of five simultaneous equations, in variables *u*_*β *_(*β *∈ Σ) and *λ*.


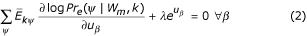



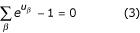


The derivation of the update formulas is somewhat involved, and is described in the Methods section. The equations are solved using Newton's method, and the solution value of *u*_*β *_is used to update the (*k*, *β*)^*th *^entry of *W*_*m*_, according to *W*_*mkβ *_= *e*^*uβ*^. Newton's method involves computation of the first and second partial derivatives of log *Pr*_*e*_(*ψ*|*W*_*m*, *k*_), as described in Methods. In practice, we found that Newton's method always converges from a single initial condition, and the convergence almost always happens within 3–5 iterations.

The time complexity of (each E-M iteration in) PhyME is O(*LKl*), where *L *is the length of the sequences, *K *is the number of species, and *l *is the length of the motif desired. (See Methods for details.)

### Results on synthetic data

We first present the results of running PhyME on synthetic data. The experimental framework is largely borrowed from Wang and Stormo [20]. In each experiment, 5 "ancestral" sequences, each of length 600 bp, are created at random, and 20 "binding sites", each of length 8, are "planted" at randomly chosen locations in these sequences. The sites are chosen such that the weight matrix formed by them has a relative entropy of *R*. Each ancestral sequence is then "evolved" by point mutations to create *K *additional "orthologous" copies, assuming a star topology (with *K *leaves) and a common "background mutation rate" *μ*_*b *_along each branch. (No insertions or deletions were included in this simulated evolution, for simplicity.) The motif instances are subjected to a common "motif mutation rate" *μ*_*m *_, which is the probability of mutation of any position in a motif. The ancestral set of sequences is then removed and the remaining *K *orthologous sets are input to the motif discovery algorithm, with one arbitrarily designated the reference species. The algorithm is made to report 3 different motifs, thereby making some allowance for false positives, especially when *R *is low. For each reported motif, its 20 best occurrences in the reference species are compared with the planted occurrences, to give a score ranging between 0 and 1. (1 represents the best possible performance; see the Methods section for details.) The score for the best of the 3 reported motifs is the "performance score" of the algorithm. The three algorithms being compared are PhyME, MEME [1], and GIBBS (Wadsworth Gibbs sampler) [24]. PhyME was run with an evolutionary tree with a star topology, the mutation rate along each branch being *μ*_*b*_. MEME and GIBBS were run on the entire data set pooled together, ignoring the orthology of sequences.

**Figure 2:**
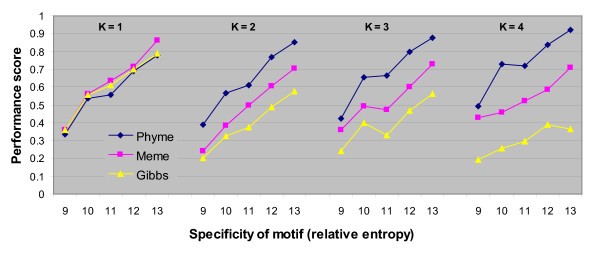
Effect of varying the number of species (*K*) on motif-finding performance. The x-axis is the relative entropy (*R*) of the planted motif. Each point is an average over 10 experiments with synthetic data. (*μ*_*b *_= 0.3, *μ*_*m *_= 0.1.)

Figure 2 shows the effect of varying *K *on performance scores of the algorithms. Note that the performance of PhyME, while similar to MEME and GIBBS for *K *= 1, improves relative to them as *K *increases. The absolute performance score of GIBBS (and of MEME, to some extent) deteriorates with increasing *K*. With more orthologous sequences, conserved stretches of background sequence may distract the algorithm from the motif occurrences. PhyME, with the additional knowledge of orthology, is able to pick out the motif better.

**Figure 3:**
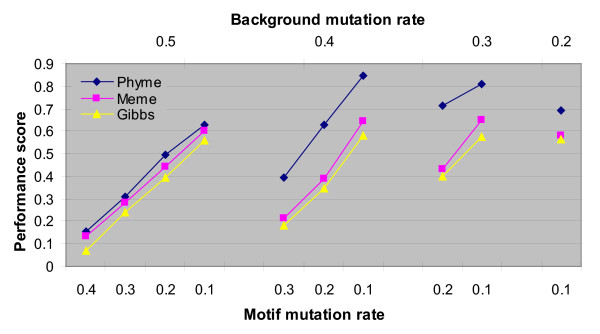
Effect of varying background and motif mutation rates (*μ*_*b *_and *μ*_*m *_respectively) on motif-finding performance. Each point is an average over 10 experiments with synthetic data. (*K *= 3, *R *= 12.)

**Figure 4:**
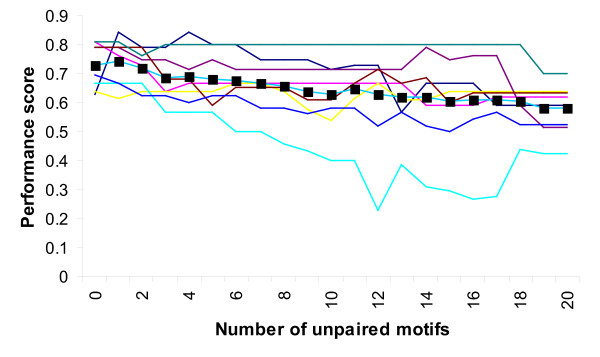
Effect of the alignment step on motif-finding performance. The x-axis shows how many of the orthologous pairs of planted motifs are artificially unpaired in the alignment step. Each solid line represents a separate experiment. The squares plot the average score over eight experiments.

Figure 3 shows the effect of varying the mutation rates. The background mutation rate *μ*_*b *_was varied from 0.2 to 0.5 and the motif mutation rate *μ*_*m *_was varied between 0.1 and *μ*_*b *_- 0.1). As per expectation, the performance of each algorithm improved with decreasing *μ*_*m *_(for a fixed *μ*_*b*_). Interestingly, as *μ*_*b *_decreases, the performance of PhyME for *μ*_*m *_= 0.1 first improves and then falls down. The initial improvement is because the alignment step is able to find more conserved blocks with diminishing background mutation rate. However, when the latter approaches the motif mutation rate, the distinction (in cross-species conservation) between motif and background becomes weaker, hence performance goes down. In another set of experiments, we examined the effect of the alignment step on the performance. Sequences were created with *K *= 2, *μ*_*b *_= 0.3, *μ*_*m *_= 0.1 and *R *= 12. After the alignment step of PhyME (in which the entire sequence was aligned as one conserved block), we artificially "unpaired" some number *n *of the planted orthologous motif occurrences, i.e., the alignment was modified so that these *n *pairs fell outside conserved blocks. This was followed by the usual motif-finding step, and the entire procedure was repeated for various values of *n*. We find a gradual degradation in performance as PhyME moves from maximum utilization of motif orthology (*n *= 0, no unpaired motifs) to minimum (*n *= 20, no motif pairs considered orthologous). (Figure 4.)

We also evaluated the effect of mis-estimates of the neutral mutation rates on performance. PhyME was run on random sequences created with experiment parameters *K *= 3, *μ*_*b *_= 0.3, *μ*_*m *_= 0.1 and *R *∈ {11, 12, 13}, and in different runs, the value of *μ*_*b *_input to PhyME ranged from 0.1 (underestimate) to 0.5 (overestimate). We observed that underestimates of *μ*_*b *_resulted in significantly greater performance degradation than overestimates of equal magnitude. (Data not shown.) For instance, using *μ*_*b *_= 0.4 instead of the true value of 0.3 made no difference to the performance, whereas using *μ*_*b *_= 0.2 resulted in 15 – 50% decline.

### Results on biological data

In the following sections, we present results of running PhyME on real data sets from yeast, fly and human. The results are compared to MEME (run by pooling orthologous sequences together), orthoMEME [15], PhyloGibbs [17], and EMnEM [18]. The latter three programs address the heterogeneous data problem directly, just as PhyME does. Another program that solves the same problem is PhyloCon [20]. PhyloCon was not evaluated in our study because we did not have a clear method to post-process its output to extract a specified number of top-scoring motifs that are non-redundant. (Our evaluations described below use the top three motifs reported by each program.)

#### Yeast data sets

We first present some examples in yeast, where sequence data from four species, *S. cerevisiae*, *S. mikatae*, *S. kudriavzevii *and *S. bayanus *was used. We performed motif-finding (with PhyME, MEME, orthoMEME, PhyloGibbs and EMnEM) on some regulons from the SCPD [25] database, which catalogues sets of co-regulated genes. For each regulon, the top *η *motif occurrences in *S. cerevisiae *reported by the algorithm (*η *being the number of known sites in *S. cerevisiae*) were examined for "matches" to the known weight matrix for that regulon's motif. (See Methods for details.) The number of matches was the performance score of the algorithm. We counted matches to the weight matrix, rather than to known sites, so that a reported motif occurrence that is very similar to the known motif (but not annotated as a site by SCPD) will not be counted as a false positive. Each algorithm reported 3 motifs (with *η *occurrences for each motif), and the results are for the best scoring motif, thereby making some allowance for false positives, such as simple repeats. Even though PhyME is implemented to handle arbitrary phylogenies, for efficiency it was run with a phylogenetic tree with a star topology, having *S. cerevisiae *at the center and the mutation rate along the branches of *S. mikatae*, *S. kudriavzevii*, and *S. bayanus *being (0.25, 0.3, 0.35) respectively. These values are based on average substitution rate per base in the corresponding pairs of species. (A more accurate tree can be derived from the work of Kellis *et al*. [12].) For multiple species data, MEME was run by pooling all sequences together. OrthoMEME was run only for the case *K *= 2 (i.e., on sequence from the two species *S. cerevisiae *and *S. mikatae*), since its current implementation can only handle two species data. The other four programs were run for *K *= 1, 2, 3, 4, in separate executions.

See Methods for details on how orthoMEME, PhyloGibbs and EMnEM were run.

**Figure 5:**
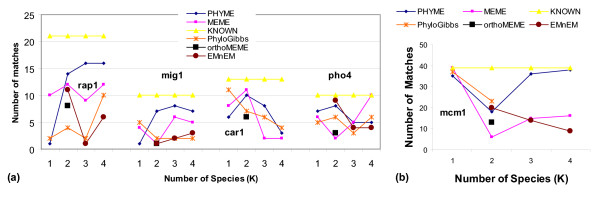
Effect of multiple species information on motif-discovery in the regulons RAP1, MIG1, CAR1, PHO4 and MCM1 in yeast. The y-axis plots the number of matches to the known motif, among the top *η *reported occurrences, where *η *is the number of known sites, plotted as "KNOWN" . Only matches in *S. cerevisiae *are considered.

Figure 5 plots the performance scores for regulons RAP1, MIG1, CAR1, PHO4 and MCM1, which show interesting results. Note how the performance of PhyME improves with *K *for RAP1 and MIG1. For CAR1, both PhyME and MEME improve from *K *= 1 to *K *= 2, and then deteriorate for higher *K*, but PhyME at *K *= 3 is still better than at *K *= 1. For PHO4, PhyME's performance first goes up (for *K *= 2) and then dips below the *K *= 1 level, whereas MEME shows best performance at *K *= 4. For MCM1, PhyME scores well (over 90% matches) for *K *∈ {1,3,4}, whereas MEME's performance degrades for *K *> 1. Thus, these examples illustrate that PhyME's approach can lead to improved motif discovery in multiple species data, and also that there may be situations when more orthologous sequences distract either algorithm from the true motif. For regulons CSRE, GCN4, MAT*α*2, URS1H, REB1, and PDR3, the performance score was high (typically over 80% matches) and largely invariant of the number of species.

Figure 5 also reports the scores of PhyloGibbs. This program has similar scores as PhyME on CAR1, PHO4 and MCM1. (It did not execute to completion for *K *= 3, 4 in MCM1.) PhyME has better scores on MIG1 and RAP1, though PhyloGibbs' scores on RAP1 with a different choice of parameters ("-G 0.7", see Methods) were similar to PhyME. (Data not shown.) We also report the scores of EMnEM in Figure 5. (The program did not execute to completion in CAR1.) This program performs well for *K *= 2 (comparable to the best scores in the data sets RAP1, PHO4 and MCM1). For *K *= 3, 4 also, EMnEM scores are comparable to PhyloGibbs. PhyME typically performs better than EMnEM (with *K *= 3, 4) for RAP1, MIG1, MCM1, and comparably for PHO4.

We find the scores of orthoMEME, as reported in Figure 5, to be lower than those of PhyME (for *K *= 2). However, we observed that in all five regulons reported, orthoMEME reported fewer than *η *occurrences in *S. cerevisiae *per motif. This is because orthoMEME was run in the "zoops" mode (zero or one motif occurrence per sequence), since the "tcm" mode (any number of occurrences per promoter) does not perform well. Thus, with the total number of predictions being fewer than *η*, orthoMEME's scores are expected to be lower than other programs even for the same level of specificity.

We suggest caution in comparing PhyME's scores directly with those of the other programs, since we lacked expertise in choosing optimal parameter settings for them. This is particularly true for EMnEM, which has several parameters for modeling the evolution of motifs, and we lacked experience in setting these parameters optimally. We clearly have more expertise at using PhyME than the other programs, and this makes the comparisons biased. Our goal in these experiments was to provide some examples of how multiple species data can be exploited to improve performance, rather than assessing the different motif finding programs available. A proper comparative assessment of these programs has to address several challenges not addressed here. Such a task was undertaken for several motif finding programs, in the work of Tompa *et al*. (unpublished). A similar assessment of the motif-finding programs in the context of the heterogeneous data problem is an important topic for future work.

#### Fly data sets

Next, we present results from fruitfly, where data from two species, *D. melanogaster *and *D. pseudoobscura*, is available. Nine different enhancers were chosen – enhancers *eve1*, *eve2*, *eve5*, *ftz3*', *gtposterior*, *hairy2*, *hairy34*, *and run1 *have binding sites for the *Kr *transcription factor, and *btdhead *has *Bcd *sites.

Well-defined weight matrices are available for both *Kr *and *Bcd *[26]. For each enhancer, the top *η *motif occurrences (in *D. melanogaster*) reported by the algorithm (*η *being the number of "strong" occurrences of the known weight matrix in *D. melanogaster *– see Methods) were examined, and the number of matches was the performance score of the algorithm. Six different motif-finding strategies were tested separately – (i) MEME_1 (MEME on single species) (ii) MEME_2 (MEME on both species pooled together), (iii) PhyloGibbs, (iv) orthoMEME, (v) EMnEM and (vi) PHYME (PhyME on both species, with *μ *= 0.5). Each strategy was required to report occurrences only in *D. melanogaster*. (See Methods for details of how orthoMEME, PhyloGibbs and EMnEM were run.)

**Figure 6:**
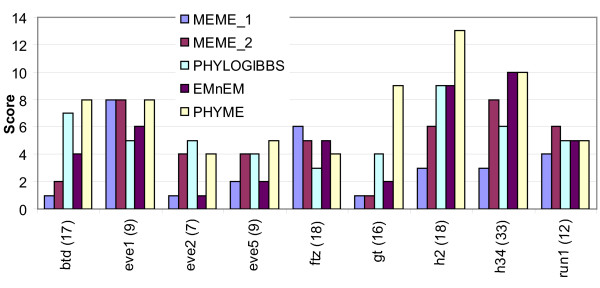
Comparison of PhyME to 1 species and 2 species MEME, and to PhyloGibbs and EMnEM, for fly enhancers. The parenthetical number next to an enhancer name is the number of strong occurrences of the known weight matrix, in the *D. melanogaster *sequence.

Figure 6 compares the scores of the different strategies for all nine enhancers. Note that either PHYME or MEME_2 performs better than MEME_1 for seven of the nine enhancers, and worse only for one (*ftz3*'), thereby making the case for using two species data. Moreover, on *btdhead*, *gtposterior *and *hairy2*, PhyME performs significantly better than MEME_2, demonstrating the advantage of using orthology information. Similarly, we find PhyME to perform better than PhyloGibbs on *gtposterior*, *hairy2 *and *hairy34*. EMnEM performs well in these data sets, scoring comparably to PhyME or PhyloGibbs, except on *btdhead*, *eve2*, and *gtposterior*, where both PhyME and PhyloGibbs perform better, and *hairy2*, where PhyME alone performs better. OrthoMEME was run in the "tcm" mode, since the "zoops" mode is not appropriate here, with several putative sites to be found in each promoter. However, orthoMEME tends not to perform well in the "tcm" mode in general, and in our tests also, its scores were poor on most of the enhancers. We thus find that PhyME is preferable to orthoMEME for cases where we expect several motif occurrences per sequence.

As in the yeast data sets, the comparison of scores between PhyME and the other programs should be interpreted with caution, since we lacked expertise in choosing optimal parameter settings for the other programs.

#### Human data sets

**Figure 7:**
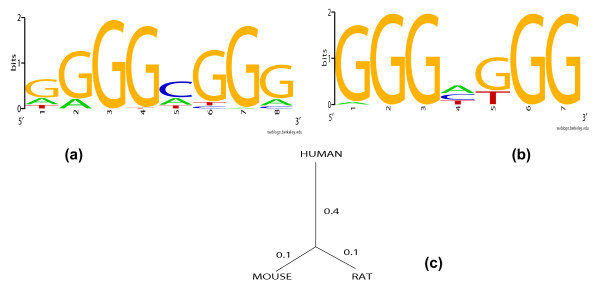
Results on the human SP1 regulon. (a) The known motif. (b) Motif reported by PhyME, using mouse and rat orthologs. (c) The phylogenetic tree used by PhyME.

Finally, we present results of running PhyME on two data sets from human, where orthology with mouse and rat was utilized. These data sets were chosen because all of 15 different motif-finding programs tested in an assessment project (Tompa *et al*., unpublished) failed to report the correct motif in them. The first set corresponds to the transcription factor SP1, a zinc-finger protein. The heterogeneous sequence data included 35 human promoters (of length 2 Kbp each), of which four have orthologous promoters from mouse and rat, 20 from mouse only, 4 from rat only, and 7 have no orthologs. Each of the human promoters is known to have at least one functional Sp1 binding site, with a total of 76 known sites overall. Figure 7a shows a "sequence logo" representation [27] of an alignment of these known sites. PhyME was run to find motifs of length 7, using the phylogenetic tree shown in Figure 7c. The second motif reported by PhyME (Figure 7b) is almost identical to the known SP1 weight matrix. The top 27 instances (in human promoters) of this motif included 16 that overlapped with known binding sites. We also ran MEME on the heterogeneous data set (pooling orthologous sequences together), and the second motif reported was a good match to SP1. However, of its 41 instances reported in human promoters, only 9 were overlapping with known sites. Moreover, when MEME was run on human promoters alone, none of the top three motifs matched the SP1 motif. Thus, PhyME showed a clear performance improvement over MEME, both in the single species run, and when the orthologous sequences were pooled together.

**Figure 8:**
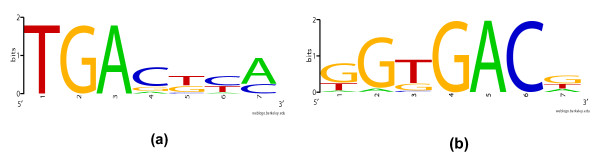
Results on the human c-Jun regulon. (a) The known motif. (b) Motif reported by PhyME, using mouse and rat orthologs.

The second data set used in our tests corresponds to the leucine zipper transcription factor c-Jun. The heterogeneous data set included 500 bp promoters for 11 human genes targeted by c-Jun, as well as orthologs from mouse and rat for 3 genes, from mouse only for 5 genes, and from rat only for the remaining three genes. PhyME was run exactly as in the previous data set. The known binding sites of c-Jun (in human) were aligned to produce a weight matrix that is shown in Figure 8a. The third ranked motif reported by PhyME is shown in Figure 8b, and we can see that its last five positions are similar to the first five positions of the known weight matrix. Of the top 13 instances of the discovered motif, 4 overlap with known binding sites of c-Jun, whereas a maximum of 9 could have been obtained. (Of the 11 known sites, 9 are in the 500 bp upstream regions used in our analysis.) We also ran MEME on the heterogeneous data set (using the pooling strategy), and none of the three best motifs reported by MEME matched the c-Jun motif. Thus, both the human data sets tested demonstrate how PhyME can improve motif discovery in typical motif finding scenarios by exploiting heterogeneous sequence data properly.

## Discussion

### Issues in algorithm design

#### Alignment step

In the alignment step, PhyME extracts blocks of high sequence similarity between the reference species and each of the other species. Motif occurrences in such locally conserved regions are deemed orthologous, an assumption well-justified by traditional interpretations of sequence alignment. Conversely, all orthologous motif occurrences are assumed to be aligned in such blocks. This assumption is not always true since there may be orthologous motif occurrences not aligned by the alignment program, but it heavily constrains the space of orthologous motif occurrences, implying greater efficiency of the search algorithm. Moreover, the assumption does not mean that "true" orthologous occurrences in unaligned regions are ignored – they are merely treated as independent occurrences. Our experiments on synthetic data (see Results) demonstrate that the performance is not very sensitive to the correct alignment of all orthologous motif pairs. The blocks computed in the alignment step have to be with respect to the reference species, but the alignment itself need not be done in a pairwise manner. A multiple alignment of all sequences may be computed (e.g., with M-LAGAN [21], using the input phylogenetic tree Ψ) and blocks between 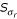 and each of the other *S*_*i *_may then be extracted. (The alignment step is implemented as a separate tool in PhyME, making it easy to switch to such alternative schemes.) Furthermore, the implementation may be modified in the future to drop the requirement of a reference species, since this requirement is not crucial to the motif finding step of PhyME. For instance, the alignment step may utilize the "Threaded Block Alignment" (TBA) program of Blanchette *et al*. [28], which completely circumvents the notion of a reference species in multiple alignments.

Once the blocks of high sequence conservation have been identified, a possible strategy is to restrict attention to motif occurrences in these blocks, assuming that all functional binding sites must be evolutionarily conserved. However, this assumption is not true even for as closely related species as *D. melanogaster *and *D. pseudoobscura*, separated by about 25–30 Myrs. An empirical study [16] on these two species revealed that a good fraction (35–40%) of occurrences of relevant motifs occur outside of locally conserved contexts, and should therefore be taken into account when discovering motifs.

#### Motif Finding

In the probabilistic process that is assumed to generate sequences, the transition probability does not depend on the previous choice(s) made during the process, meaning that the HMM is of zeroth order, nor on the position in the sequence, meaning that any information about spatial distribution of motifs is ignored. The model, unlike that of MEME, does not fragment the sequence into all *l*-length words to be treated independently. Rather, it parses the sequence into a series of non-overlapping occurrences of the motif and background.

The evolutionary model described by Formula 1 applies only to phylogenies having a star topology. The general case of arbitrary tree topology is described in Methods. In Formula 1, if *μ*_*σ *_is small (as for very closely related species), then finding different bases in orthologous positions has low probability *Pr*_*e*_(*ψ*|*W*, *k*), even if their frequency in *W *is the same. This mirrors the intuition that mutations in locally conserved regions of closely related species are evidence against a binding site residing there. For largely diverged species (i.e., if *μ*_*σ*_~1, ∀ *σ*), *Pr*_*e*_(*ψ*|*W*, *k*) reduces to the product of the individual bases' probabilities. It is worth emphasizing here that the weight matrix *W *being searched by the algorithm is assumed to be unchanged over the entire phylogenetic tree (including the ancestor).

The neutral mutation rates (probabilities) along each branch of Ψ are input by the user and not trained during E-M. Training them on input data may cause overfitting, producing values that are largely inconsistent with the known evolutionary distances. The work of Moses *et al*. [18] studies this issue, and finds that it is more important to use correct phylogenetic relationships, e.g., an appropriate evolutionary tree, than to use accurate mutation rates.

Note that the evolutionary model used by PhyME comes into play only in Equations 2 as the term *Pr*_*e*_(*ψ*|*W*_*m*_, *k*). Other models of evolution, e.g., F81 [29], can be incorporated into PhyME by simply using the appropriate formulation of this term, as long as the derivatives of log *Pr*_*e*_(*ψ*|*W*_*m*_, *k*) can be computed efficiently.

## Conclusions

We have developed a new algorithm, PhyME, that detects motifs in heterogeneous sequence data by integrating two important aspects of a motif's significance – overrepresentation and cross-species comparison – into one probabilistic score. We have evaluated different aspects of the algorithm on synthetic data, and demonstrated on some biological data sets that the new approach improves motif detection.

## Methods

### The evolutionary model

The evolutionary model makes the following assumptions: (i) Nucleotides in an aligned position are evolved from a common ancestor. (ii) The weight matrix applies to the common ancestor and to all descendants, a reasonable assumption given the propensity of DNA binding domains of proteins to evolve slower than cis-regulatory modules. (iii) All positions evolve independently, at equal rates, and the probability of fixation of a mutation *α *→ *β *at position *k *is proportional to the weight matrix entry of *β *at that position. Suppose we are given a phylogenetic tree Ψ, with the species {*σ*_1_, *σ*_2_, .... *σ*_*K*_} at the leaves. Let the vector *ψ *= (*s*_1_, *s*_2_, ... *s*_*K*_), where *s*_*σ *_is the nucleotide from species *σ *in the (single position) alignment *ψ*. The term *Pr*_*e*_(*ψ*|*W*, *k*) denotes the probability of observing *ψ *at position *k *when sampling from weight matrix *W*. For each node *j *of the tree, except the root, let *μ*_*j *_be the probability of a base in the parent species of *j *having mutated (under neutral evolution) in species *j*. Also, let *ψ*_*j *_be the vector formed by elements of *ψ *that correspond to leaf nodes descended from node *j*. Let *C*(*j*) denote the set of children of node *j *and let *r *be the root of the tree. Then, we can write (using the model assumptions):


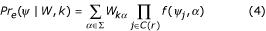


where *f*(*ψ*_*j*_, *α*) denotes the probability of observing *ψ*_*j *_given that the base at the parent of *j *is *α*. For a leaf node *σ*, this can be written as 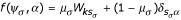, from the model assumptions. (*δ*_*ij *_= 1 if *i *= *j*, and 0 otherwise.) For an internal node *j *(except root *r*), the expression is :


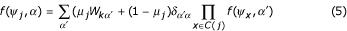


For the special case where Ψ has a star topology, Equation 4 reduces to Equation 1.

### Training parameters in a HMM

Given a sequence *S *and a set of position weight matrices {*W*_*i*_}, the objective function to be maximized is *F*(*S*, *θ*) = log(*Pr*(*S*|*θ*)/*Pr*(*S*|*θ*_*b*_)), where *Pr*(*S*|*θ*) is the probability of generating the sequence *S *using the parameters *θ*, and *θ*_*b *_represents the parameter values that only allow the background motif *W*_*b *_to be used by the HMM. The sequence *S *can be written as *ψ*_1_*ψ*_2 _... *ψ*_*L*_, where each *ψ*_*i *_is either a single base or an alignment of orthologous bases at a single position. *θ *includes the weight matrices *W*_*i *_and their transition probabilities *p*_*i*_. Since *Pr*(*S*|*θ*_*b*_) depends only on *W*_*b*_, which is assumed constant, we shall outline how to maximize log *Pr*(*S*|*θ*), following the description in [23]. A parse of the sequence *S *in terms of the *W*_*i *_(i.e., the series of motifs chosen in the successive steps of the generative probabilistic process) is denoted by *T*.

We thus have


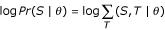


The maximization is iterative, with the *t*^*th *^iteration computing a model *θ*^*t *+ 1 ^that improves the objective function from the current model *θ*^*t*^. In classical E-M fashion, let us define a function *Q*(*θ*|*θ*^*t*^) as


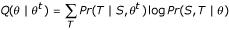


It is easily shown that log *Pr*(*S*|*θ*) - log *Pr*(*S*|*θ*^*t*^) ≥ *Q*(*θ*|*θ*^*t*^) - *Q*(*θ*^*t*^|*θ*^*t*^). Thus, if we maximize *Q*(*θ*|*θ*^*t*^) over all *θ*, we shall always improve upon log *Pr*(*S*|*θ*^*t*^), or remain there if the local maximum has been reached. Let *A*_*i*_(*T*, *S*) be the number of times *W*_*i *_occurs in the parse *T *of *S*. Also, let *E*_*ikψ*_(*T*, *S*) denote the number of times that the alignment *ψ *is emitted (sampled) at the *k*^*th *^position of the matrix *W*_*i*_, in parse *T *of *S*. Let *l*_*i *_denote the length of *W*_*i*_. Then we have


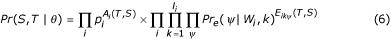


which gives us


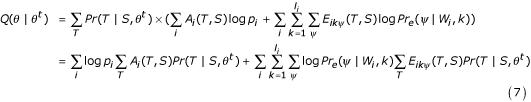


Note that the only the first term in this expression depends on *p*_*i*_, and only the second term depends on *W*_*i*_. Hence, we maximize each of these terms independently, with respect to the appropriate free parameters. We first maximize the term


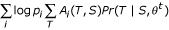


Note that 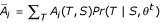 is the average number of occurrences of *W*_*i *_in *S *over all parses *T*.

Thus the term to maximize is 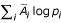, and this is maximized when


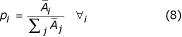


Next, we maximize the second term:


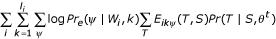


Again, note that 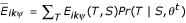 is the average number of times that the alignment *ψ *is sampled at the *k*^*th *^position of the matrix *W*_*i *_while generating *S*, the average being over all parses *T*. Thus, the term to maximize is 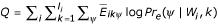. We first note that in our case, there is a single weight matrix *W*_*m *_to be trained. Hence, we need to maximize *Q *with respect to *W*_*m*_. We can do this maximization with respect to each column *k *independently. Let *W*_*kβ *_denote the (*k*, *β*)^*th *^entry of *W*_*m*_. Thus, for each *k *= 1 ... *l*, we need to maximize *Q *with respect to *W*_*kβ *_(*β *∈ Σ), with the constraint Σ_*β *_*W*_*kβ *_= 1. Using Lagrangian multiplier *λ*, the objective function becomes *Q *+ *λ *(Σ_*β *_*W*_*kβ *_- 1).

Transforming to log variables *u*_*β *_= log *W*_*kβ *_to ensure that the *W*_*kβ *_remain positive during optimization, we then have the following necessary conditions for optimality (in addition to the constraint 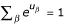) :


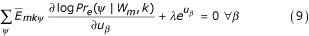


We therefore have a system of five equations (including the constraint) in the variables *u*_*β *_(∀*β *∈ Σ and *λ*. Denoting the vector of these five variables by **u**, we solve this system of equations using Newton's iterative method. Let us write the above system of equations as **F**(**u**) = **0**, where **F**(**u**) = [[*f*_*β*_], *f*_*λ*_], with *f*_*β *_being the left side of Equation 9, and 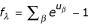. Newton's method uses the update relation:

Δ**u **= -(**J**(**u**))^-1^**F**(**u**)

where Δ**u **is the change in **u **in the current iteration and **J **is the Jacobian matrix of **F**. The important terms in the computation of **F **and **J **are the first and second partial derivatives of log *Pr*_*e*_(*ψ*|*W*_*m*, *k*_) with respect to the *u*_*β *_variables. For this purpose, we need to compute *Pr*_*e*_(*ψ*|*W*_*m*, *k*_) and its first and second partial derivatives. Computation of *Pr*_*e*_(*ψ*|*W*_*m*, *k*_) uses the formulas 4 and 5. The partial derivatives can be computed recursively (over the tree Ψ) by using the chain rule of differentiation. These recursive computations are implemented in a bottom-up manner, so as to avoid redundant computations. Newton's method uses **F **and **J **to iteratively compute new values of **u**, until convergence. The Jacobian matrix **J **in our case is not positive definite, hence Newton's method is not guaranteed to converge. However, in practice, we found the method to always converge from a single initial seed. Upon convergence, the log variables *u*_*β *_are transformed back to *W*_*kβ *_= *e*^*uβ*^. The procedure is repeated for each *k *= 1 ... *l*, and *W*_*m *_is then updated with the new values. This update, along with that given by Equation 8, is used iteratively to improve *F*(*S*|*θ*) until the local maximum is reached, as indicated by a very small change in its value.

### Time complexity

The E-step computes 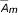, 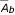 and 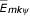, for *k *= 1 … *l*, ∀ *ψ*. The Forward-Backward algorithm is run once, in *O*(*LKl*) time, where *L *is the total length of the input sequences, *K *is the number of species, and *l *is the length of the motif *W*_*m*_. (This time complexity assumes that nodes in the phylogenetic tree have a fixed maximum degree.) Thereafter, 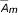, 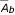 are computed in *O*(*L*) time, and all the 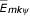 are computed in one scan of the input, expending *O*(*Ll*) time.

The M-step runs Newton's method to solve a system of equations, once for each column of *W*_*m*_. Each run of Newton's method goes through a small number (3–5) of iterations. Each iteration computes the first and second partial derivatives of log *Pr*_*e*_(*ψ*|*W*_*m*, *k*_) Each of these derivatives can be computed in *O*(*K*) time, where *K *is the number of species (since |*ψ*| ≤ *K*) Hence, **F **and **J **can be computed in *O*(*LK*) time, where *L *is the total length of the sequence. Hence, Newton's method takes *O*(*LK*) time, and is run *l *times, for an overall time complexity of *O*(*LKl*) for the M-step.

Thus, the running time of (each E-M iteration in) PhyME scales linearly with the length of the sequences, the length of the motif desired, and the number of species.

### Implementation details

PhyME is implemented in C++ for Linux, and the source code will be made freely available at . The current implementation runs in a few minutes (on a workstation) for typical applications with total sequence length ~10000 bp, 2–4 species, and motif length of ~10.

PhyME uses the LAGAN alignment tool of Brudno *et al*. [21] for the alignment step. After alignment, the ungapped blocks extracted are required to be at least 10 bp long, and have at least 70% identity. PhyME is implemented to handle an arbitrary phylogenetic tree Ψ relating the input species.

The E-M algorithm is guaranteed to converge only to a local optimum. To address this problem, the motif-finding step is executed a fixed number of times, each time using a randomly chosen substring of the input sequence as the "seed" to initialize *W*_*m*_, and truncating the E-M procedure after a small number of iterations. The seed with greatest score *F*(*S*, *θ*) among these runs is then used to run the E-M to convergence and the trained motif is reported, along with all its instances with posterior probability above a certain threshold. To find more motifs, PhyME masks out the central base of each of these instances. Optionally, the user may specify nsites, the expected number of occurrences of each of the desired motifs. In such a case, PhyME turns off training of the parameter *p*, and uses a fixed value computed from nsites. Similarly, an option maxsites specifies the maximum number of occurrences expected.

PhyME considers occurrences on both strands by introducing a new weight matrix *W*_*r*_, and an associated transition probability *p*_*r*_, in the HMM parameters. The weight matrix is constrained to be the reverse complement of *W*_*m*_. The model has a fixed bias of planting the motif in one orientation versus the other, and this bias is trained from the data. PhyME also has the option of capturing local correlations in background nucleotide composition. To implement a *κ*^*th *^order Markov background, PhyME uses a special background weight matrix that is of length 1 but uses the knowledge of the previous *κ *bases generated to determine the emission probabilities of the next base.

### Performance score in experiments with synthetic data

We use the following score for measuring the performance of a motif-finding algorithm on synthetic data. Let *S *= {*S*_1_, *S*_2_, ... *S*_*n*_} be the set of *n *input sequences. For any motif *m*, let *I*_*mi *_be the set of positions in sequence *S*_*i *_that are occupied by an occurrence of *m*. We know the occurrences of the planted motif *m*^*k*^, and are evaluating the motif *m*^*r *^reported by an algorithm. The performance score Φ is defined as follows:


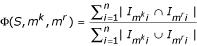


In other words, it is the number of positions, over all sequences, where occurrences of the known and reported motifs overlap, divided by the total number of positions at which the known *or *the reported motif occurs. Note that if the reported occurrences exactly concur with the known occurrences, the score is 1, and when the reported and known occurrences have no position in common, it is 0.

### Details of experiments with biological data sets

#### Yeast

The genes regulated by each transcription factor are listed in SCPD. For each such "regulon", the known sites and the known weight matrix were extracted from SCPD. Also, 800 bp long upstream sequences of the genes in each regulon were extracted (for *S. cerevisiae*) from the RSA-Tools web site [30]. Orthologous promoters in the other yeast species were obtained from Cliften *et al*. [11]. Let *η *be the number of known binding sites in *S. cerevisiae*. The input to the motif finding algorithm consisted of the sequences from *S. cerevisiae *and their orthologs from one or more of the other species, depending on *K*. (In addition to *S. cerevisiae*, we used *S. mikatae *for *K *= 2, *S. mikatae *and *S. kudriavzevii *for *K *= 3, and *S. mikatae*, *S. kudriavzevii *and *S. bayanus *for *K *= 4.) The length of the motif was also input to each program. Each algorithm was made to report 3 motifs, and for each motif, the top *η *reported occurrences in *S. cerevisiae *were examined. For each such occurrence, the logarithm of the probability of sampling it from the known weight matrix was computed, and a *z*-score of this logarithm was obtained. If the *z*-score was above 3, the occurrence was called a "match". To allow for slight offsets in the reported motif, each reported occurrence was padded with 3 bp of its context, on either side.

PhyME was run with the *maxsites *option set to *η*, and MEME was run with the same option set to *ηK*. We also experimented with running MEME with the *nsites *parameter set to *ηK*. OrthoMEME was run with a zeroth order Markov background, in the "zoops" mode, with expected number of sites between 0.8*η ("minsites") and 1.2*η ("maxsites"). PhyloGibbs was run with mutation probability 0.7 ("-G 0.3") for all species, and was asked to report three motifs (three "colors") each with 1.5 × *η *occurrences ("-I") initially. A 3^*rd *^order Markov background ("-N 3") trained on the full complement of yeast promoters was used, as with PhyME and MEME. The "loose align" option ("-D 1") and the "stop after anneal" option ("-X") were used. These options were suggested by an author of PhyloGibbs (Rahul Siddharthan, personal communication). We experimented with a different value for the mutation probability ("-G 0.7"), with no improvement, except in the RAP1 regulon. EMnEM was run with default parameters, the motif length being input through the "-w" parameter. Phylogenetic trees were derived from each input promoter, using the fastDNAML software of Olsen *et al*. [31]. The alignments were done using the MLAGAN program of Brudno *et al*. [21]. In separate runs, we also tried non-default values of the parameters "-p" (relative rate of motif to background; default 0.5, also tried 0.25) and "-m" (evolutionary model; default Jukes-Cantor, also tried F81). The expected number of instances of each motif per sequence ("-e") was set to *η*/*n *and *η*/*n *+ 1 in separate runs, where *n *is the number of input promoters. For each data set, and for each value of *K*, we took the best scoring choice of parameters. This was done to give some advantage to EMnEM, since we lacked expertise in choosing optimal parameter values.

#### Fly

The locations of cis-regulatory modules involved in body-patterning of the early embryo in *D. melanogaster *were obtained from [26], and their sequences were extracted from BDGP [32]. The evaluation procedure was identical to that in yeast, with the following difference. Since there is no complete list of verified sites in the enhancers, we first scanned the sequences (in *D. melanogaster*) with the known weight matrix, and counted matches, by the same measure as above. This count was the value of *η *used in the experiment. An extra complication in the fly data is caused by the fact that each enhancer typically contains sites for multiple transcription factors. We restricted our tests to the factors Kr and Bcd, because their weight matrices are of better quality than others. Moreover, for each enhancer, we chose to test with the transcription factor with most putative sites (matches to its weight matrix).

OrthoMEME was run as in the yeast data sets (see above), except that the "tcm" mode was used now. PhyloGibbs was also run as in the yeast data sets, except that we used a mutation probability of 0.5 ("-G 0.5"), and a 2^*nd *^order Markov background ("-N 2"), trained on non-coding regions in fly. We also experimented with a higher value of the mutation probability, and tried specifying the initial number of occurrences per motif ("-I") differently, with no clear improvement. EMnEM was run with the Jukes-Cantor evolutionary model ("-m 0") and with the relative rate of motif to background ("-p") set to 0.5 and 0.25 in separate runs. The expected number of motifs was set to *η *and 1.5 × *η *in separate runs. The best performing choice of parameters was used for each data set.

#### Human

The genes comprising each regulon were obtained from TRANSFAC [33]. Mouse and rat orthology information for human genes was obtained from Homologene [34]. Human, mouse and rat promoters were obtained from the UCSC Genome Browser [35].

## Authors' contributions

All authors participated in initial discussions leading to the key idea of using Expectation-Maximization and a phylogenetic model to search in a weight-matrix space. SS designed the algorithm details, derived the E-M calculations, implemented and tested the program, and drafted the manuscript. All authors contributed to, read and approved the final manuscript.
